# *Schistosoma japonicum* in Ethiopia: Is there a need for tuning schistosomiasis surveillance in Africa?

**DOI:** 10.1371/journal.pntd.0013425

**Published:** 2025-08-13

**Authors:** Yamral Abatneh, Getaneh Alemu, María Flores-Chávez, Banchamlak Tegegne, Belay Bezabih, Tesfa Demelie, Kefale Ejigu, Jose Miguel Rubio, Isabel de Fuentes, Melaku Anegagrie, Aranzazu Amor Aramendia

**Affiliations:** 1 Medical Laboratory Department, Shumabo Health Center, Amhara Regional Health Bureau, Bahir Dar, Ethiopia; 2 Department of Medical Laboratory Science, College of Medicine and Health Sciences, Bahir Dar University, Bahir Dar, Ethiopia; 3 Reference and Research Laboratory in Parasitology, National Center for Microbiology, Instituto de Salud Carlos III, Madrid, Spain; 4 Mundo Sano Foundation, Madrid, Spain; 5 Amhara National Regional State, Public Health Institute, Bahir Dar, Ethiopia; Federal University of Agriculture Abeokuta, NIGERIA

## Abstract

**Background:**

Schistosomiasis is a communicable disease of public health importance in Africa, where *Schistosoma mansoni* is the most prevalent species. The determinant of the geographic distribution of *Schistosoma* spp. is a snail, the intermediate host, specific for each species affecting human beings. The accurate identification of cases is essential for the success of control programs in endemic areas. The current work presents a case of *Schistosoma japonicum* in a woman living in Ethiopia.

**Case presentation:**

In Ethiopia, in an area endemic for *S. mansoni*, a woman attended a health center, because of joint and abdominal pain. She had never travelled out of the country. She was living in the shore of the Blue Nile River, in Bahir Dar city, which receives a large Chinese population for years. A stool sample was analyzed using Kato Katz, saline concentration and polymerase chain reaction techniques, indicating a co-infection by *S. japonicum* and *S. mansoni*.

**Conclusions:**

This study raises awareness about the event, previously noticed, that populations migration is linked to new scenarios of schistosomiasis species. The role of local microscopists becomes of great importance for accurate detection and epidemiological surveillance.

## Author summary

The geographical distribution of the *Schistosoma* spp. is determined by the habitat of the snail intermediate host, specific for each species. Authors present a case of *Schistosoma japonicum* diagnosed in Ethiopia, in a patient with no history of travel out of the country, living in an *S. mansoni* endemic area that, from more than 10 years, receives regularly population from China. The finding implies the presence of *Oncomelania hupensis*, the intermediate snail for *S. japonicum*, not noticed in Africa. The fact that this snail is amphibious, can survive for long periods, tolerates a wide range of temperature and keeps long time its ability for transmitting schistosomiasis after being carried to non-permissive areas would support the hypothesis that this finding might not be an isolated case, and that transmission of *S. japonicum* could be going unnoticed, in the context of a continuous migratory movement of Chinese population to the African continent. Awareness on that possibility will avoid adding challenges for schistosomiasis control programs in endemic African countries that receive migrants from *S. japonicum* prevalent areas.

## Introduction

Schistosomiasis is a neglected tropical disease caused by parasitic flatworms (blood flukes) of the genus *Schistosoma* [1]. In 2023 the World Health Organization reported over 250 million people affected globally, more than 90% of them living in sub-Saharan Africa, where the infection is a public health problem [2]. Of the seven species that infect the human, three are most common: *S. mansoni*, in Africa, Middle East, Caribbean and South America; *S haematobium*, in Africa and Middle East; and *S. japonicum,* in China, Indonesia and the Philippines. *S. intercalatum*, *S. guineensis* and *S. mekonyi* have much lower prevalence, the first two restricted to Central Africa, and the third along the Mekong River, in South-East Asia [3]. Finally, human infection by *S. malayensis* has been described in Malaysa, but in a very low prevalence [4]. Infective larvae grow in fresh-water snails, the intermediate host, which is specific for each *Schistosoma* spp. Therefore, the schistosomiasis geographical distribution is defined by the habitat range of the snails. *S. mansoni* and *S. haematobium* infect the aquatic *Biomphalaria* and *Bulinus* spp. snails respectively, while *S. japonicum* infects the amphibious snail *Oncomelania hupensis* [5] Human is the definite host, infected by the cercaria stage through penetrating the skin, when contacting with contaminated water [6]. The infection affects mainly poor, rural communities, but has recently spread to urban and peri-urban zones [7]. Also, schistosomiasis introduction and transmission in non-endemic areas has been noticed due to population movement [8].

With over 126.5 million people, Ethiopia is the second most populous nation in Africa and one of the poorest [9]. Schistosomiasis is among the neglected tropical diseases (NTDs) prioritized by the Ministry of Health of the country, where two species are found: *S. mansoni* widely distributed, and *S. haematobium*, more delimitated in foci in the Rift Valley region. In 2020 the population at risk for schistosomiasis was estimated to be 53.3 million, almost half of the country’s population. Endemic areas implement preventive chemotherapy (PC) control programs, but reinfection is common, and the provision of water, sanitation and hygiene services along with snail control programs are gaps that challenge the success of the interventions [10]. Here, we report a case of *S. mansoni* and *S. japonicum* co-infection in Ethiopia.

## Case report

### Study area

Bahir Dar city is the capital of the Amhara Region in the north-western of Ethiopia. The city is situated between the Blue Nile River and Tana Lake basins, at 1,800 m above sea level. It comprises an urban nucleus and a surrounding rural area. The location is under the influence of three different seasons, which impact *Schistosoma* infections: rainy, from June to September; spring, from mid-October until the end of December; and dry, the rest of the year. The area is of low endemicity for *S. mansoni* and highly endemic for soil-transmitted helminths (STH) [11].

### Case presentation

**Fig 1:**
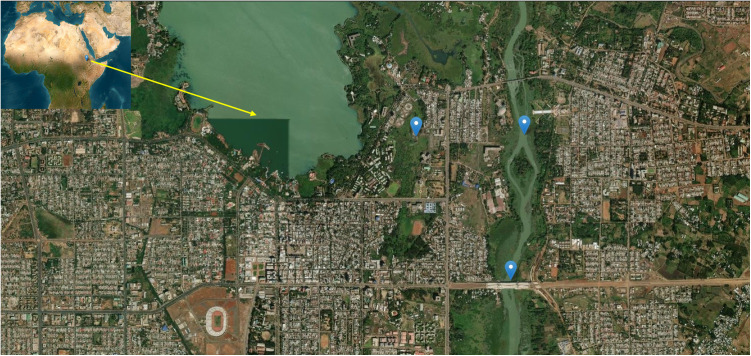
Bahir Dar in the south tip of Tana Lake, Amhara Region, north-western Ethiopia. Location of the health center (arrow on the top left, Lat; 11⁰ 36’ 00” N, Lon: 037⁰ 23’ 59” E), 0.3 Mi far from Tana Lake, 0.5 Mi far from Blue Nile River (arrow on the top right, Lat; 11⁰ 36’ 00” N, Lon: 037⁰ 24’ 33” E) and 2,4 Mi far from the new bridge over the river (arrow at the bottom, Lat; 11⁰ 35’ 16” N, Lon: 037⁰ 24’ 28” E). Top left of the figure: Location of Bahir Dar in the horn of Africa. Base Layer obtained from the U.S. Geological Survey, National Geospatial Program. (https://earthexplorer.usgs.gov/) (https://www.usgs.gov/faqs/what-are-terms-uselicensing-map-services-and-data-national-map).

In October 2022, a 66-year-old woman, attended the Shumabo health center in the urban area of Bahir Dar. She was living in a neighborhood about 200 m from the Blue Nile River (Fig 1). She was complaining about joint pain for four days duration. She had a previous history of asthma. At the time of the consultation, she also referred to cough, anorexia, nausea, vomiting and abdominal cramps, but she was not able to precise the duration of symptoms. Previously, she had treated herself through traditional medicine with leaves of neem tree (*Azadirachta indica*), because of the abdominal cramps, but she could not remember the exact time and duration of the treatment. Regarding her epidemiological background, she used to collect pipe or tap water for domestic activities; she did not mention recent contact with fresh water or surface water, she had no history of agricultural, livestock or irrigation activities, nor washing clothes in natural fresh-water sources. She had no travel history outside of Ethiopia.

### Case management

A physical examination was done with no remarkable findings. The woman was treated with salbutamol, as the cough was linked to her previous history of asthma; albendazole, the empiric treatment of abdominal cramps in an STH endemic area; and indomethacin, for the joint pain.

## Methods

### Ethics statement

Written consent was obtained by authors from the patient, after receiving relevant explanation.

### Laboratory procedures

**Fig 2:**
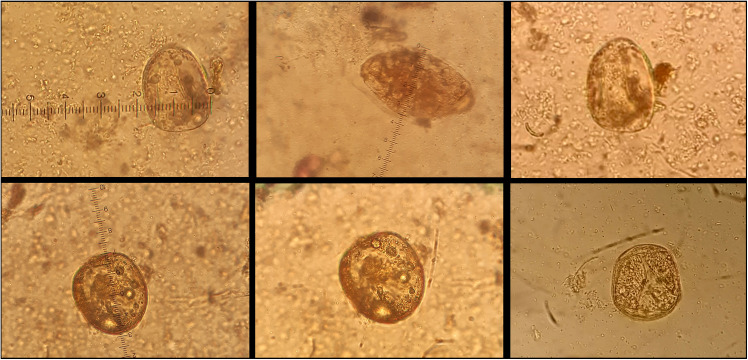
*Schistosoma* spp. eggs found in the sample by a concentration with saline solution (25X objective): *Schistosoma japonicum,* at the top left, top right, bottom left, bottom center and bottom right pictures (average size: 56 µm x 74 µm); *Schistosoma mansoni,* at the top, in the center picture.

A stool sample was sent to the laboratory of the health center for parasitological examination with a direct wet mount test, which revealed structures presumably identified as eggs of *S. japonicum.* The finding was confirmed by a thick smear on a microscopic slide, processed by the Kato Katz technique, for the detection of eggs in stool, stained with malachite green. The diagnosis was corroborated in the reference laboratory of the region, the Amhara Public Health Institute in Bahir Dar. A stool sample was sent to the National Center for Microbiology, Instituto de Salud Carlos III, in Madrid, Spain. A microscopic examination was done, after a concentration with saline solution with the Bioparaprep MINI system (Leti Diagnostics, Barcelona, Spain) which is based on a modification of Ritchie’s method, with filtering been enhanced by a filtration-concentration process [12]. Both eggs of *S. japonicum* and *S. mansoni* were identified (Fig 2). The load of *S. japonicum* ova was higher, being on average 10 eggs of S*. japonicum* per field, while only 3 eggs of *S. mansoni* were identified in all the preparation. Two qualitative real time polymerase chain reaction (PCR) assays were performed for *S. japonicum* and *S. mansoni* identification. An automated DNA extraction, by using the QIAcube (QIAGEN, Hilden, Germany) instrument was done, from 200 mg of the sample. For *S. japonicum* identification a PCR assay targeting the NADH dehydrogenase I mitochondrial gene was used [13], with primer sequences SjND1FW, forward: 5′-TGR TTT AGA TGA TTT GGG TGT GC-3′, and SjND1RV reverse: 5´-AAC CCC CAC AGTCAC TAG CAT AA-3′. Briefly, 2 μl of DNA, 1 μl of each primer (10mMol), 10 μl of a QUANTIMIX HotSplit Probes kit master mix (Biotools, Madrid, Spain), and 0.15 μl of SYBR green (Merck, Darmstadd, Germany) were included in a final volume of 20 μl. The PCR cycling conditions were as follows: 2 min initialization at 50ºC, 10 min denaturation at 95ºC, 40 cycles of 15 s denaturation at 95ºC, 60 s annealing at 60ºC, 90 s and a final extension at 72ºC. Melt curve analysis was performed for each PCR. The hybridization temperature for the primer set was 66.25ºC. For *S. mansoni* identification the amplification of the 28S rDNA region was utilized using previously described primers [14] SmF 5′-GAG ATC AAG TGT GAC AGT TTT GC-3′ and SmR 5′-ACA GTG CGC GCG TCG TAA GC-3′. PCR was performed in a final volume of 20 μl. containing 5 μl of DNA, 0,2 μl of each primer (10mMol), and 12,5 μl of a QUANTIMIX HotSplit Probes kit master mix (Biotools, Madrid, Spain). The thermal profile consisted of 3 min at 94°C followed by 35 cycles of 30 s at 94°C, 20 s at 65°C, and 20 s at 72°C with a final extension at 72°C for 7 min. Amplifications and detection were carried out using a Rotor-Gene Q RT-PCR cycler (Qiagen, Hilden, Germany).

## Results

The sample was found to be positive for both PCR, confirming a co-infection by *S. japonicum* and *S. mansoni*.

### Follow up

The patient was treated with praziquantel, and the PCR one year later was negative.

## Discussion

Ethiopia has one of the highest prevalence schistosomiases in sub-Saharan Africa [15]. The infection is prioritized in the NTD Master Plan for public health control [10]. However, the country has historically focused on a case-based treatment of laboratory-confirmed patients. The lack of standardized studies and gaps in the mapping challenge PC programs [16]. To our knowledge, this is the first local *S. japonicum* case in Africa. The presence of a specific snail in is essential for allowing *Schistosoma* spp. to complete their life cycle [1]. In Ethiopia, there are no reports of *O. hupensis,* the only intermediate snail host of *S. japonicum*. However, some snails have the capability to invade new areas and the potential introduction of schistosomiasis in non-endemic areas, related to this has been alerted [17]. In Bahir Dar there are large settlements of Chinese population, working in industrial activities [18], road construction [19] or education-integration training programs in the university [20]. At the time this case was reported, a bridge over the Blue Nile River was under construction (Fig 1). The project started on August 2019, being managed by a Chinese company, which has been in Ethiopia for more than two decades [21]. In a widespread model in Chinese construction industry in Africa, the workers were living in an isolated dormitory labor compound [22] built in the shore of the Blue Nile River, alongside the bridge under construction. Their close and frequent contact with fresh water has been illustrated by the gradual rise in *S. haematobium* or *S. mansoni* cases observed in field workers returning from Africa to China [23]. In such context, we consider two plausible hypotheses for this finding, either the transport (from China to Ethiopia) of infected snails or the transport of uninfected snails, lately infected by *S. japonicum*, released in the river by workers suffering an unnoticed infection in China. For explaining those two hypotheses, we should point out that *O. hupensis* is not an aquatic but an amphibious snail that can be carried out of water. Moreover, even if infected, it can survive for 12 months or longer and may tolerate cold temperatures, down to 0°C [5]. Moreover, its ability for transmitting schistosomiasis does not change after migrating from permissive to non-permissive areas for at least 13 years and even this ability has been highlighted as a crucial issue for reducing the risk of *S. japonicum* spread [24]. Therefore, the transport of infected snails, either in personal baggage or in big containers and construction materials, and the contamination of the Blue Nile River water are a reasonable hypothesis of the finding of *S. japonicum* in this location of Ethiopia. The second possibility would be the local infection of imported *Oncomelania* snails from workers releasing *S. japonicum* into the Blue Nile River. Even more, there is a possibility of eggs or larvae of the Oncomelania snails developed once in Ethiopia until adult snails and then be infected [25]. The distance to the river, a factor with strong correlation to *O. hupensis* infestation probability [26] will not to be an obstacle as the workers were living on the shore of the Blue Nile River. However, further studies would be necessary, as there was no research for snail populations and more studies are required for supporting effective control activities, including the spatial distribution of intermediate host and the status of the infection in freshwater snails, as there is a strong influence of land-use and environmental factors, including high scale construction activities, on the abundance, occurrence, and infectivity of several species of snails identified in the Blue Nile and Lake Tana basins. [27] As far as we know, there is no notice of *O. hupensis* cases in the country, nor have studies been designed for the specific detection of this species. The convenience of further research focusing on this snail in the future should be properly evaluated. We should point out that, together with the ability of *O. hupensis* to survive, the difficulties in achieving the control of schistosomiasis japonica are also attributed to its high number of definitive host reservoirs [28]. Of more than 40 species identified to be naturally infected by *S. japonicum* approximately one third have a significant role in its transmission, mainly rodents, mainly rodents, (*Rattus rattus*), goats (*Capra hircus*), common in Africa including Ethiopia, and specifically in Bahir Dar and the Blue Nile basin [29,30]; water buffalo (*Bubalus bubalus*) present in some African countries [31]; and dogs (*Canis familiaris*) and cats (*Felis domestica*) widespread in Africa [32,33].

The co-infection with *S. mansoni*, which is endemic in the area, proves the contact with fresh water of the patient, even though it was not clearly identified in the anamnesis. This contact could have occurred not only on the river or in the lake but also in other water sources common in the rainy season. During the rainy season (the patient was diagnosed at the end), ponds and streams develop very fast everywhere, resulting in the displacement of the snail population following the water flow with the subsequent increase of schistosome infections [34]. The process of urban expansion that mostly affects peri-urban land in Bahir Dar in recent years [35] has also been linked to new foci of schistosomiasis [7].

About symptoms, *S. mansoni* clinical manifestations are comparatively milder than the *S. japonicum* because the adult *S. japonicum* female produces ten times eggs per day greater than *S. mansoni* [36]. This fact is coherent with the microscopic findings, with a higher load of *S. japonicum* ova. Abdominal cramps, anorexia and coughing reported by the patient are compatible with both *S. mansoni* and *S. japonicum* infection [37]. As for joint pain, this symptom has been associated with *S. haematobium* and *S. mansoni* [38]; muscle pain has been described in *S. japonicum* infection [39] but not joint injury.

We should point out that China has been the Africa largest trading partner for the last 15 consecutive years. For instance, in 2022, the top five countries receiving Chinese workers were the Democratic Republic of Congo, Algeria, Egypt, Nigeria, and Angola [40] accounted for 42% of all Chinese workers in Africa. In all but Algeria schistosomiasis is a public health issue and PC for control is required [41]. A case report does not allow generalized to an entire population but, from a public health view, we set if this case has been an isolated event in a city in Ethiopia, or if this could be possible in other areas receiving workers from *S. japonicum* endemic areas. The awareness and expertise of technicians in local laboratories will be essential for confirming a broader hypothetical scenario, as the eggs could have been confused with more common ones in the context, such as *Ascaris lumbricoides*. A limitation was the lack of an optimal sequencing: the homology of the amplicon sequence with the one obtained from the GenBank was 60%. Of note, not significant similarity *S. mansoni* was found. The sample quality was poor when molecular diagnosis was performed in Spain and when a new sample was collected after treatment the result was negative. This limitation highlights the convenience to provide full capacity for molecular diagnosis in endemic areas, supporting the diagnosis in real time. Likewise, even though the ability of *O. hupensis* for surviving allows us to hypothesize in the context of a migrant population, snail surveys were not conducted, and this will be important for further investigation. China has actively participated in Africa’s public health governance during the past 60 years through the China-Africa Health Cooperation and since 2013 with the Belt and Road Initiative for global infrastructure development and international cooperation, which includes supporting African countries in the control of infectious diseases and strengthen public health systems [42]. As the role of Ethiopian health workers for schistosomiasis control programs is essential, the possibility of the hypothesis stated in this paper gives a precise field for China commitment on capacity-building strategies in Ethiopia (e.g., training for microscopists and other diagnostic strategies) to scale up surveillance.

## Conclusions

This case highlights that unexpected infections can occur due to the movement of workers who live in uncontrolled conditions. A case of *S. japonicum* diagnosed in Ethiopia, in a patient with no travel history out of the country lets to hypothesize that this finding might not be an isolated case, in the context of a continuous migratory movement of Chinese population in Africa. An awareness on that possibility will help schistosomiasis control programs in African countries receiving migrants from *S. japonicum* endemic areas. The role of local microscopists becomes of great importance for diagnosis as well as for epidemiological surveillance. Also, serology, available in different formats, could be an affordable method for screening [43] The convenience of an active surveillance system for travelers and materials shipped from *S. japonicum* endemic areas could be evaluated in hot spots, addressing this problem, in line with China’s global health initiatives [44] and in the frame of the road map for NTDs [45], for avoiding and anticipating unexpected challenges, for the 2030 targets of the schistosomiasis control programs in Africa.
